# Unplanned intensive care unit admission after elective colon cancer resection: population-based registry study

**DOI:** 10.1093/bjsopen/zraf178

**Published:** 2026-03-09

**Authors:** Sofia Dahlberg, Tomas Vedin, Ingvar Syk, Emma Larsson, Niklas Nielsen, Henrik Bergenfeldt

**Affiliations:** Department of Surgery, Helsingborg Hospital, Helsingborg, Sweden; Department of clinical Sciences, Lund University, Lund, Sweden; Department of Surgery, Malmö and Institution of Clinical Sciences Malmö, Malmö, Sweden; Department of Surgery, Malmö and Institution of Clinical Sciences Malmö, Malmö, Sweden; Department of Perioperative Medicine and Intensive Care, Karolinska University Hospital and Department of Physiology and Pharmacology, Karolinska Institutet, Stockholm, Sweden; Department of Anaesthesiology, Helsingborg Hospital and Department of Clinical Sciences, Lund University, Lund, Sweden; Department of Surgery, Lund and Department of Clinical Sciences Lund, Department of Surgery, Lund University, Skåne University Hospital, Lund, Sweden

**Keywords:** surgical complications, colon cancer survival, critical care

## Abstract

**Background:**

The incidence, aetiology, and outcome of unplanned intensive care unit admission after elective colon cancer surgery remain unclear. This study investigated the incidence of, and factors associated with, unplanned intensive care unit admission following elective colon cancer resection in Sweden.

**Methods:**

This nationwide retrospective registry study included adult patients undergoing elective colon cancer resection with curative intent in Sweden between 2010 and 2019. Patients with distant metastases, or rectal or appendiceal tumours were excluded. Data from the Swedish Colorectal Cancer Registry and the Swedish Intensive Care Registry were analysed. Patients not requiring intensive care unit admission served as controls.

**Results:**

Of 23 891 patients, 1343 (5.6%) required unplanned intensive care unit admission. These patients were older, had more co-morbidities, and were more likely to undergo open surgery and receive permanent stomas. Patients requiring surgical reintervention accounted for 43% of intensive care unit admissions and were identified later (day 5 *versus* day 1), had longer duration of stay in the intensive care unit (3 *versus* 1 day), and had worse outcomes than those with non-surgical complications, despite being younger, with less co-morbidity. Intensive care unit admission was linked to a higher unadjusted mortality rate at 30 days (13.9 *versus* 0.6%), 1 year (24.2 *versus* 4.6%), and 3 years (40.0 *versus* 15.3%). Laparoscopic surgery was associated with reduced intensive care unit admissions (odds ratio 0.59, 95% confidence interval 0.50 to 0.69) and lower 3-year mortality (odds ratio 0.79, 0.72 to 0.86), even after adjusting for patient- and surgery-related factors.

**Conclusion:**

Unplanned intensive care unit admission was associated with increased short- and long-term mortality. Patients who had surgical reinterventions leading to intensive care unit admission were admitted later and had poorer outcomes than those with non-surgical complications, highlighting the need for earlier recognition and tailored postoperative monitoring strategies.

## Teaser

This nationwide Swedish study analysed 23 891 patients who underwent elective colon cancer surgery between 2010 and 2019, and found that that 5.6% required unplanned admission to the intensive care unit. These patients had higher mortality rates and more complications, particularly when surgical reintervention was needed.

## Introduction

Surgical resection is the core of curative management for colon cancer. Morbidity and mortality rates following elective colon cancer resection have declined owing to advances in surgical technique, preoperative optimization, patient selection, and improved postoperative care^1,2^. However, a significant percentage of patients still develop complications that affect both short- and long-term survival, irrespective of tumour stage^3^. In-hospital mortality rates of 2–5% have been reported after elective colon resection^4^ and many postoperative deaths have been preceded by admission to the intensive care unit (ICU).

Postoperative intensive care unit (ICU) admission is sometimes planned before operation, or occurs as a result of patient deterioration while in a lower-acuity setting. Unselected ICU admission after elective colon resection is generally not recommended^5,6^. However, in certain patients, such as elderly and frail people, planned overnight ICU admission may decrease the risk of postoperative adverse events, attributed to closer monitoring and earlier intervention than in a regular surgical ward^7^. Studies^8,9^ investigating the aetiology of postoperative ICU admission and its association with patient outcome after elective colon cancer resection are scarce. Commonly, ICU admission is considered a binary measure, and no distinction is made according to the precipitating cause of ICU admission. Hence, the impact of unplanned postoperative intensive care after elective colon cancer resection remains largely unknown.

The primary aim of this study was to evaluate the incidence of, and factors associated with, unplanned ICU admission following elective colon cancer surgery. The secondary aim was to analyse short- and long-term survival.

## Methods

### Study design

This was a retrospective, observational multicentre study based on registry data from the Swedish Colorectal Cancer Registry (SCRCR) and the Swedish Intensive Care Registry (SIR). This study was designed and conducted in accordance with the STROBE guidelines^10^. Ethical approval was obtained from the Swedish Ethical Review Authority (2021-02448).

### Registries

The SCRCR comprises data on consenting patients diagnosed with colon cancer since 2007 and rectal cancer since 1995 (https://scrcr.se/). The registries cover 98–99% of all colorectal cancers in Sweden, compared with compulsory registration in the National Cancer Registry. The SCRCR is linked to the Swedish population registry, from which survival data are retrieved. The SIR was launched in 2001 and is a national quality registry of patients admitted to ICU. It has complete coverage of Swedish ICUs (https://www.icuregswe.org). Available data in the SIR include baseline demographics, variables included in the Simplified Acute Physiology Score (SAPS3), and variables relating to treatment given in the ICU.

### Inclusion process

Patients undergoing elective colon cancer resection between January 2010 and December 2019 were identified from the SCRCR and included in the study. Included patients were matched with the SIR to identify postoperative ICU admissions. Exclusion criteria were non-surgical management of the primary tumour, palliative surgical intent, synchronous metastases (M1) at diagnosis, tumour location in the rectum or appendix, and planned ICU admission. Planned ICU admissions are defined by the SIR as admissions that are known by the ICU for ≥ 12 h. In this study, admissions were considered to have been planned before operation if they were registered as a planned admission and occurred on the day of surgery.

### Study protocol

The postoperative period was defined as a 2-week interval from the day of surgery throughout the second postoperative week. Patients requiring postoperative ICU admission constituted the ICU group, and those who received treatment in a regular surgical ward only served as the control group. The ICU group was further stratified into admissions depending on whether surgical reintervention was needed or not. Surgical reintervention is defined by the SCRCR as any unplanned postoperative laparotomy, surgical procedures in the operating room or in the ICU, or percutaneous radiological interventions, corresponding to Clavien–Dindo grade IIIa–b. The rationale for this distinction is that complications requiring surgical intervention are usually more serious than those handled without surgical intervention, and usually make the patient more ill, and more likely to require intensive care.

### Data analysed

The following SCRCR data were extracted: age, sex, American Society of Anesthesiologists (ASA) fitness grade, surgical approach (open *versus* minimally invasive), anatomical resection, perioperative blood loss, intraoperative bowel perforation, duration of operation, ostomy decision, complications, and complications requiring reintervention. The SCRCR classifies complications as cardiovascular, infectious, neurological, surgical or other.

Details regarding the ICU admission were retrieved from the SIR, comprising length of ICU stay, reasons for ICU admission, whether the admission was planned or unplanned, non-invasive ventilation, invasive ventilation, continuous renal replacement therapy (CRRT), vasoactive support, SAPS3, and estimated mortality risk (EMR). Possible reasons for admission are cardiovascular, gastrointestinal, respiratory, renal, neurological, other, and observation.

SAPS3 is a risk stratification score used to predict death in patients admitted to the ICU. It is based on three areas: patient co-morbidities, such as chronic conditions and diseases; data regarding the ICU admission, such as reason for admission and surgical status; and the patient’s physiological derangement within 1 hour before to 1 hour after ICU admission^11^.

### Statistical analysis

Data with a normal distribution are presented as mean(standard deviation) whereas skewed continuous and ordinal data are presented as median (interquartile range). Categorical data are presented as numbers with percentages. Student’s *t* test was used for hypothesis testing of normally distributed data, Wilcoxon rank-sum test for skewed and ordinal data, and χ^2^ test for categorical variables. Normality was assessed using the Kolmogorov–Smirnov test.

Univariable and multivariable logistic regression analyses were used to identify factors associated with unplanned ICU admission and death. The regression variables were selected based on clinical judgement and results from previous studies^9,12^. Survival was estimated using the Kaplan–Meier method, and the log rank test was used to assess differences between groups.

The significance level was set to 0.05. Data were processed using Stata^®^ BE version 18 (StataCorp, College Station, TX, USA).

## Results

### Study population characteristics

**Fig. 1:**
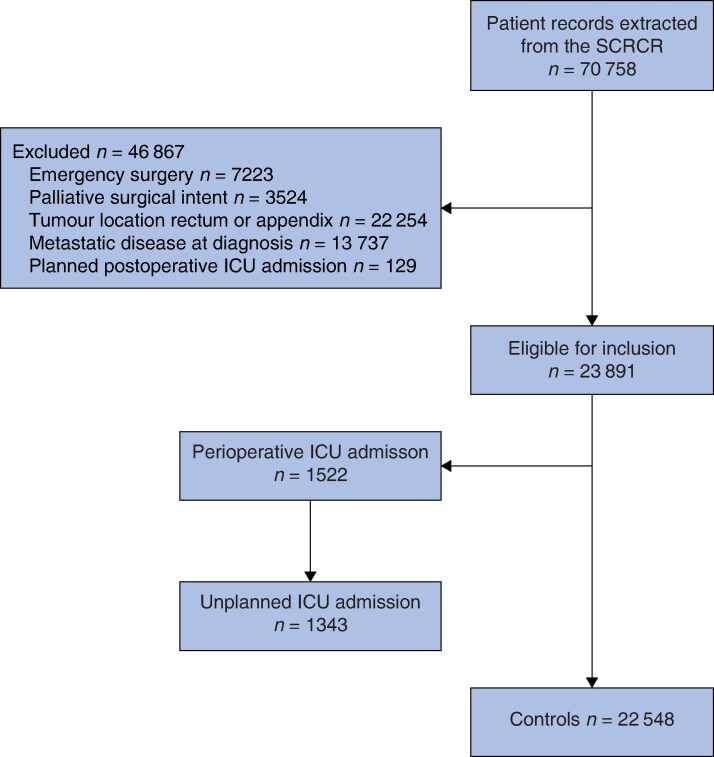
Study flow chart SCRCR, Swedish Colorectal Cancer Registry; ICU, intensive care unit.

In total, 70 758 patient records were extracted from the SCRCR, of which 23 891 fulfilled the inclusion criteria and were included in the study. A total of 1343 patients (5.6%) had unplanned postoperative ICU admissions and constituted the ICU group. The remaining 22 548 patients not admitted to ICU comprised the control group (*Fig. 1*).

### Patient and perioperative characteristics

Clinical and demographic characteristics of the ICU group and controls are summarized in *Table 1*. Patients requiring intensive care were predominantly men (58 *versus* 48.5%) and were older (77 *versus* 74 years) with higher ASA grades than controls. Among 569 patients with an ASA grade of IV, 17% had unplanned postoperative ICU admissions. Tumour stage and the range of operations were broadly similar, although the ICU group was less likely to be operated with a minimally invasive technique (12.1 *versus* 24.1%), and a larger proportion received permanent ostomies. Differences in operating time and blood loss were moderate.

**Table 1:** Clinical and demographic characteristics of patients who underwent elective resection of stage I–III colon cancer, stratified by need for postoperative ICU admission

	ICU (*n* = 1343)	Controls (*n* = 22 548)
**Patient characteristics**		
Age (years), median (i.q.r.)	77 (71–83)	74 (66–80)
Missing	0 (0%)	0 (0%)
Sex		
Male	780 (58.0%)	10 945 (48.5%)
Female	563 (42.0%)	11 603 (51.5%)
Missing	0 (0%)	0 (0%)
Body mass index (kg/m^2^), median (i.q.r.)	26.0 (23.1–29.9)	25.6 (23.1–28.7)
Missing	58 (4.3%)	705 (3.1%)
ASA fitness grade		
I	52 (3.9%)	2746 (12.2%)
II	513 (38.2%)	12 113 (53.7%)
III	659 (49.1%)	6821 (30.3%)
IV	95 (7.1%)	474 (2.1%)
V	0 (0%)	4 (0.02%)
Missing	23 (1.7%)	390 (1.7%)
Tumour stage*		
pT1–3 N0	757 (56.4%)	12 496 (55.4%)
pT1–3 N1–2	326 (24.3%)	5956 (26.4%)
pT4 N0–2	245 (18.3%)	3781 (16.8%)
Missing	14 (1.0%)	315 (1.4%)
**Surgical details**		
Surgical approach		
Open	1070 (79.7%)	14 855 (65.9%)
Laparoscopic	162 (12.1%)	5433 (24.1%)
Robot-assisted	26 (1.9%)	831 (3.7%)
Laparoscopic converted to open	79 (5.9%)	1309 (5.8%)
Type of surgery		
Hartmann’s operation	40 (3.0%)	347 (1.5%)
High anterior resection	59 (4.4%)	1093 (4.9%)
Ileocaecal resection	16 (1.2%)	95 (0.4%)
Right hemicolectomy	677 (50.5%)	12 176 (54.0%)
Local excision	1 (0.07%)	21 (0.09%)
Other	11 (0.07%)	86 (0.4%)
Transverse colon resection	29 (2.1%)	331 (1.5%)
Left hemicolectomy	175 (13.0%)	2237 (9.9%)
Sigmoid colon resection	227 (16.9%)	5185 (23.0%)
Missing	1 (0.07%)	145 (0.%)
Permanent ostomy	153 (11.4%)	1293 (5.7%)
Missing	7 (0.5%)	189 (0.8%)
Perioperative blood loss (ml), median (i.q.r.)	150 (50–400)	100 (40–200)
Missing	42 (3.1%)	791 (3.5%)
Duration of operation (minutes), median (i.q.r.)	186 (141–255)	172 (133–223)
Missing	26 (1.9%)	430 (1.9%)
Intraoperative perforation	33 (2.5%)	269 (1.2%)
Missing	14 (1.0%)	223 (01.0%)
Type of perforation		
Iatrogenic	11 (0.8%)	86 (0.4%)
Spontaneous	2 (0.2%)	43 (0.2%)
Unspecified	4 (0.3%)	21 (0.09%)

Values are *n* (%) unless otherwise stated. *Staging based on postoperative pathological report. ICU, intensive care unit; i.q.r., interquartile range. ASA, American Society of Anesthesiologists.

### ICU admission

**Fig. 2:**
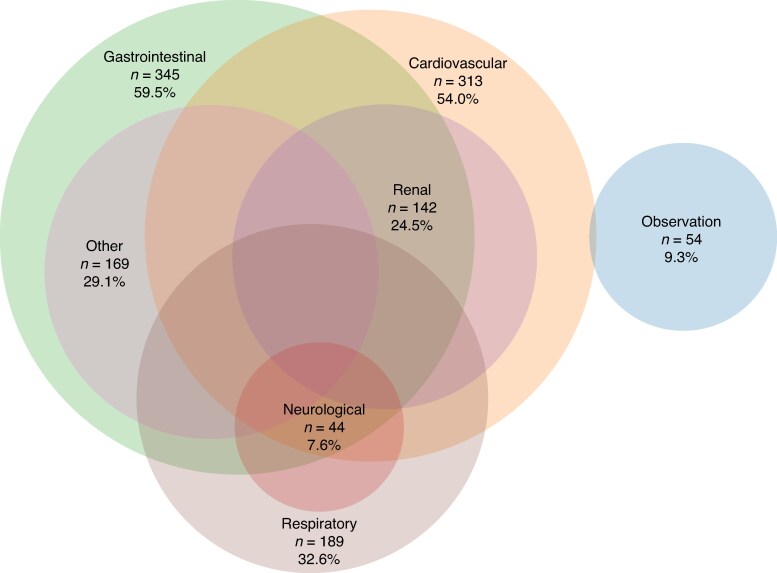
Reasons for unplanned postoperative ICU admission in surgical reintervention group ICU, intensive care unit.

**Fig. 3:**
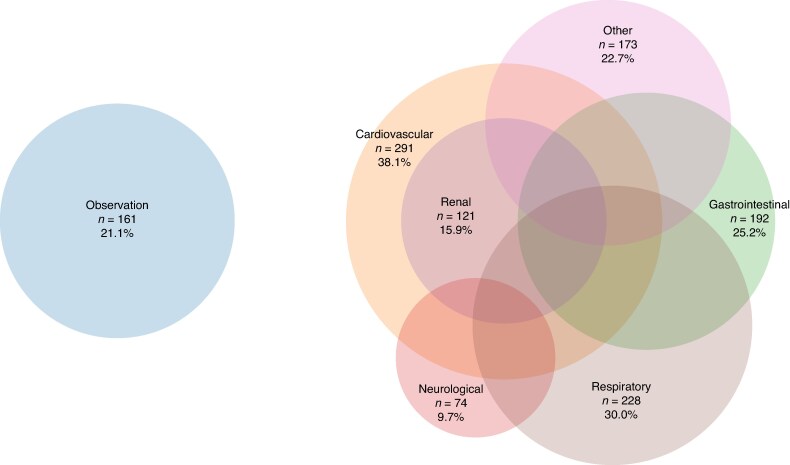
Reasons for unplanned postoperative ICU admission in group with no surgical reintervention ICU, intensive care unit.

Reasons for ICU admission are shown in *Figs 2* and *3*. The reasons for admission were multifactorial and the majority of patients had multiple organ systems affected.

Patients in the surgical reintervention group were younger and had lower ASA grades (*Table 2*). They were admitted later in relation to their primary surgery, and needed more invasive ventilation and CRRT compared with non-surgical admissions. Patients in the surgical reintervention group had a higher SAPS3 score and EMR at admission. Both ICU and hospital stay were longer for patients in the surgical reintervention group compared with the group without surgical reintervention (*Table 2*).

**Table 2:** ICU admission details

	Unplanned ICU admission		P*
	Surgical reintervention (*n* = 580)	No surgical reintervention (*n* = 763)	
Age (years), median (i.q.r.)	76 (69–82)	78 (72–83)	< 0.001†
**ASA fitness grade**			< 0.001
I	29 (5.0%)	23 (3.0%)	
II	248 (42.8%)	266 (34.9%)	
III	270 (46.6%)	389 (51.0%)	
IV	24 (4.1%)	71 (9.3%)	
V	0 (0%)	0 (0%)	
Missing	9 (1.6%)	14 (1.8%)	
**Sex**			0.001
Male	365 (62.9%)	414 (54.3%)	
Female	215 (37.1%)	349 (45.7%)	
**ICU interventions**			
Non-invasive ventilation	114 (19.7%)	125 (16.4%)	0.122
Invasive ventilation	288 (49.7%)	161 (21.1%)	< 0.001
CRRT	58 (10.0%)	19 (2.5%)	< 0.001
**SAPS3 score, median (i.q.r.)**	64 (56–73)	58 (50–66)	< 0.001†
Missing	19 (0.03%)	18 (0.02%)	
SAPS3 EMR (%), median (i.q.r.)	38 (30–47)	32 (24–40)	< 0.001†
Length of ICU stay (days), median (i.q.r.)	3 (1–7)	1 (1–2)	< 0.001†
Interval from primary surgery to ICU admission (days), median (i.q.r.)	5 (2–8)	1 (1–3)	< 0.001†
**Vasoactive drugs**	223 (38.5%)	244 (32.0%)	0.009
Missing	19 (3.3%)	18 (2.4%)	
Total length of stay (ICU + ward) (days), median (i.q.r.)	22 (14–35)	11 (7–17)	< 0.001†
**Mortality**			
30 days	91 (15.7%)	96 (12.6%)	0.104
1 year	161 (27.8%)	164 (21.5%)	0.008
3 years	240 (41.4%)	297 (39.0%)	0.363

Values are *n* (%) unless otherwise stated. ICU, intensive care unit; i.q.r., interquartile range; ASA, American Society of Anesthesiologists; CRRT, continuous renal replacement therapy; SAPS3, Simplified Acute Physiology Score; EMR, estimated mortality risk. *χ^2^ test, except †Wilcoxon rank-sum test.

### Factors associated with unplanned postoperative ICU admission

In multivariable logistic regression analysis, factors associated with unplanned ICU admission included surgical reintervention (odds ratio (OR) 8.07, 95% confidence interval (c.i.) 6.58 to 9.89), cardiovascular complications (OR 6.60, 5.39 to 8.08), ASA grade IV (OR 4.74, 3.14 to 7.16), and infectious complications (OR 3.51, 2.95 to 4.18). Laparoscopic surgery was associated with a lower rate of ICU admission (OR 0.59, 0.50 to 0.69) (*Table S1*).

### Survival analysis

Unplanned ICU admission was associated with poorer short- and long-term survival compared with those in controls. The 30-day mortality rate was 13.9% (187 deaths) for the ICU group compared with 0.6% (131) for controls (*P* < 0.001). This trend was consistent over time, with unadjusted 1-year mortality rates of 24.2 *versus* 4.6%, and 3-year mortality rates of 40.0 *versus* 15.3%, respectively (*P* < 0.001) (*Table 3*). In multivariable analysis, unplanned ICU admission and cardiovascular complications were among the factors associated with 30-day mortality (*Table S2*). These variables were also associated with 3-year mortality, although ASA grade IV had the strongest association (*Table 4*).

**Table 3:** Follow-up and survival of patients who underwent elective resection of stage I–III colon cancer, stratified by need for unplanned postoperative ICU admission

	ICU (*n* = 1343)	Controls (*n* = 22 548)	*P*
**Length of hospital stay (days), median (i.q.r.)**	15 (8–25)	6 (4–9)	< 0.001†
Missing	16 (1.1%)	152 (0.7%)	
**Planned adjuvant treatment**	140 (10.4%)	4638 (20.6%)	< 0.001
Missing	481 (35.8%)	7384 (32.8%)	
**Unadjusted mortality**			
30 days	187 (13.9%)	131 (0.6%)	< 0.001
1 year	325 (24.2%)	1040 (4.6%)	< 0.001
3 years	537 (40.0%)	3443 (15.3%)	< 0.001

Values are *n* (%) unless otherwise stated. ICU, intensive care unit; i.q.r., interquartile range. *χ^2^ test, except †Wilcoxon rank-sum test.

**Table 4:** Factors associated with 3-year mortality, estimated by multivariable logistic regression analysis

	Odds ratio	
	Univariable analysis	Multivariable analysis
Age (per year)	1.07 (1.07, 1.07)	1.06 (1.05, 1.06)
Male sex	1.13 (1.05, 1.21)	1.19 (1.10, 1.29)
**ASA fitness grade**		
I	1.00 (reference)	1.00 (reference)
II	1.94 (1.65, 2.27)	1.23 (1.04, 1.46)
III	4.74 (4.05, 5.54)	2.33 (1.95, 2.77)
IV	11.69 (9.36, 14.59)	4.57 (3.57, 5.85)
Body mass index > 30 kg/m^2^	0.98 (0.89, 1.06)	0.94 (0.85, 1.04)
Perioperative blood loss > 500 ml	1.61 (1.47, 1.76)	1.34 (1.20, 1.50)
Laparoscopic surgery	0.63 (0.58, 0.68)	0.79 (0.72, 0.86)
Perioperative perforation	1.73 (1.33, 2.25)	1.24 (0.92, 1.69)
Duration of operation (per minute)	1.00 (0.99, 1.00)	1.00 (0.99, 1.00)
Cardiovascular complication	3.95 (3.40, 4.58)	1.98 (1.66, 2.36)
Infectious complication	2.37 (2.10, 2.67)	1.48 (1.28, 1.71)
Neurological complication	3.42 (2.26, 5.17)	1.68 (1.045, 2.69)
Surgical complication	1.59 (1.46, 1.75)	1.17 (1.021, 1.34)
Other complication	1.62 (1.43, 1.83)	1.07 (0.93, 1.24)
Surgical reintervention	1.92 (1.72, 2.15)	1.1 (0.98, 1.39)
Tumour stage	1.73 (1.66, 1.80)	1.86 (1.77, 1.95)
Unplanned ICU admission	3.70 (3.30, 4.15)	2.06 (1.78, 2.38)

**Fig. 4:**
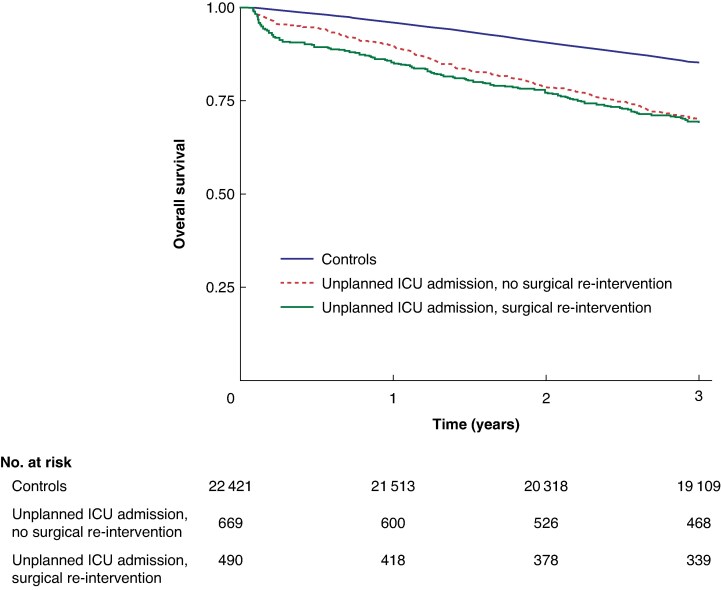
Three-year survival following elective surgery for patients with stage I–III colon cancer, stratified by need for postoperative ICU care Patients with < 30 days’ survival or follow-up were excluded from the analysis. ICU, intensive care unit.

Values in parentheses are 95% confidence intervals. ASA, American Society of Anesthesiologists; ICU, intensive care unit;

No statistically significant difference was noted in 30-day mortality depending on the need for surgical reintervention (15.7 *versus* 12.3%; *P* = 0.104). However, the 1-year mortality rate was higher in the surgical reintervention group (27.8 *versus* 21.5%; *P* = 0.008) whereas 3-year mortality rates were similar in the two groups (41.4 *versus* 39.0%; *P* = 0.363) (*Table 2*). A Kaplan–Meier curve detailing 3-year mortality is shown in *Fig. 4*.

### Complications

Complications were more common in the ICU group, irrespective of complication type, and subsequently the hospital stay was longer (15 *versus* 6 days). In the ICU group, 76.2% had a specified complication registered in the SCRCR compared with 21.6% in the control group. The proportion of surgical complications in need of reintervention was higher in the ICU group than in the control group. Surgical complications were the most common type of complication and anastomotic leakage the most common single cause for surgical reintervention (*Table 5*).

**Table 5:** Postoperative complications in patients who underwent elective resection of stage I–III colon cancer stratified by need for postoperative ICU admission

	ICU (*n* = 1343)	Controls (*n* = 22 548)
**Patients with at least one postoperative complication**	1022 (76.2%)	4864 (21.6%)
Missing	10 (0.8%)	74 (0.3%)
**Type of complication**		
Infection	328 (24.4%)	1042 (4.6%)
30-day mortality	73 (22.3%)	24 (2.3%)
Cardiovascular	261 (19.5%)	478 (2.1%)
30-day mortality	75 (28.7%)	53 (11.1%)
Neurological	20 (1.5%)	74 (0.3%)
30-day mortality	4 (20.0%)	6 (8.1%)
Surgical	582 (43.4%)	2550 (11.3%)
30-day mortality	84 (14.4%)	32 (1.3%)
Other	299 (22.3%)	1265 (5.6%)
30-day mortality	48 (16.1%)	16 (1.3%)
**Surgical complication in need of reintervention**	578 (43.1%)	1261 (5.6%)
30-day mortality	91 (6.8%)	22 (0.1%)
**Reason for surgical reintervention**		
Anastomotic leak	297 (22.1%)	469 (2.1%)
30-day mortality	43 (14.5%)	9 (1.9%)
Infection	52 (3.9%)	99 (0.4%)
30-day mortality	14 (26.9%)	3 (3.0%)
Wound dehiscence	115 (8.6%)	405 (1.8%)
30-day mortality	21 (18.3%)	4 (1.0%)
Other	168 (12.5%)	340 (1.5%)
30-day mortality	31 (18.5%)	8 (2.4%)
Interval between primary surgery and reintervention (days), median (i.q.r.)	6 (3–8)	7 (4–10)

Values are *n* (%) unless otherwise stated. ICU, intensive care unit; i.q.r., interquartile range.

The pattern of complications registered in the SCRCR differed somewhat depending on whether any surgical reintervention was required or not. The surgical reintervention group had a higher proportion of complications related to the surgery itself (86 *versus* 11%). Conversely, the rate of cardiovascular complications was higher in the group with no surgical reintervention (23 *versus* 14%). Similar distributions were observed for neurological, infectious, and other complications.

## Discussion

This registry-based study analysed the incidence, aetiology, and outcome in patients who had an unplanned ICU admission following elective colon cancer resection. The most important findings were that unplanned ICU admission was associated with increased short- and long-term mortality compared with rates in controls, and that patients admitted to ICU owing to surgical complications requiring reintervention were diagnosed later in the postoperative course than those who did not need surgical intervention, and required longer ICU and in-hospital stays. Although those requiring surgical reintervention were younger and had less co-morbidity, they had similar or higher short- and long-term mortality rates compared with those who did not have surgical reintervention.

The proportion of unplanned ICU admissions was 5.6%, with an associated 30-day mortality rate of 13.9%. This is in line with the wide range in previous studies^8,12–16^, which reported postoperative ICU admission rates ranging from 3 to 25% with in-hospital mortality rates of 11–28% depending on patient selection. Common risk factors include pre-existing cardiopulmonary disease, ASA grade III or higher, male sex, and older age, and were confirmed in the present study. In contrast to the present work, most previous studies did not discern between planned and unplanned ICU admissions, observation-only *versus* invasive measures, or the precipitating cause of critical illness.

Some studies have suggested that intraoperative microcirculation may be predictive of postoperative complications such as myocardial infarction^17^, renal failure^18^, and anastomotic leaks^19,20^. It is plausible that suboptimal haemodynamics in the early postoperative period might increase the risk of complications that become clinically evident days later. Another important factor is perioperative fluid management, and promoting a normovolaemic state to reduce the risk of cardiopulmonary complications, postoperative ileus, and oedema-related anastomotic complications^21^. Postoperative observation in a high-dependency unit or ICU could mitigate this by closer monitoring, earlier intervention, and a wider range of therapeutic options to optimize fluid homeostasis and end-organ perfusion. This strategy is supported by previous studies^7,22^ suggesting that planned ICU admission after colorectal cancer surgery might be beneficial in at-risk populations.

It is hypothetical whether any of the unplanned ICU admissions in this study could have been avoided by a higher level of care immediately after surgery and, as resources are finite, the challenge of patient selection remains.

Patients requiring surgical reintervention had similar or higher mortality rates than those admitted to ICU for other reasons, despite being younger and healthier, measured by ASA grade. These patients were admitted to the ICU later in the postoperative period and were in worse condition as measured by SAPS3 on arrival in the ICU, and required invasive ventilation, vasopressors, and renal replacement therapy more frequently.

Previous data support different temporal distributions between surgical and non-surgical postoperative complications. It has been noted that non-surgical complications tend to occur and be treated on postoperative days 1–3^23,24^, whereas surgical complications are diagnosed after a median of 7 days^25^. The question arises whether this is the natural course or whether there is a delay in the diagnosis and treatment of surgical complications, as these patients seemed to be worse off upon ICU arrival and had substantially longer recovery times. This finding further stresses the need for improving routines to discover complications at an early stage, and considerable efforts have been made to find sensitive predictors^26–32^. Reoperation within 2 days of suspicion of complication has been shown to decrease the need for intensive care, shorten hospital stay, and also to be associated with a higher chance of laparoscopic reintervention and fewer postoperative computed tomograms^33^. Unfortunately, none of the registries in this study hold data enabling analysis of whether the surgical complications were simply more severe or whether there was an element of failure to rescue involved. However, the excessive increase in mortality demonstrated in this study warrants further research to better understand what could be attributed to the postoperative level of care, and to what extent surgeon-, patient-, and tumour-related factors affect patient outcome.

It is worth noting that the ICU group received adjuvant treatment less frequently than controls, which naturally would affect their long-term outcome; however, this information was missing in about one-third of patients, thus limiting the interpretation of this variable. Several studies^34,35^ have shown that postoperative complications negatively affect both long-term survival and disease-free survival in patients with colon cancer. Comparisons between studies are hampered by methodological heterogeneity, but proposed mechanisms include increased risk of cancer recurrence, the influence of underlying co-morbidities, and immunological alterations triggered by severe illness. Overall, these findings highlight the substantial survival benefits associated with preventing serious postoperative complications.

Laparoscopic surgery was associated with fewer unplanned ICU admissions and a lower 3-year mortality rate even after adjusting for key confounders. However, this finding must be interpreted cautiously as the results likely reflect a strong selection bias, as patients selected for laparoscopy may have had less complex disease and lower perioperative risk. Despite adjustment for factors such as ASA grade and tumour stage, residual confounding cannot be excluded. Nevertheless, the data align with previous studies suggesting that laparoscopy may reduce severe postoperative complications by limiting surgical trauma^36–38^, which in turn could contribute to improved long-term survival^39^.

Taken together, the results point to the need for identifying improved routines to detect and treat complications early on and, not least, implement measures that decrease the risk of complications, such as minimally invasive approaches, goal-oriented fluid regimens, and maybe also planned ICU care in selected patients.

The major strength of this study is the combination of two nationwide registries allowing surgically oriented data to be analysed together with comprehensive records from the ICU. This enables a more detailed account of the true incidence, aetiology, and outcome in patients admitted to the ICU following elective colon resection. In contrast to most previously published data, this study provides information on whether the ICU admission was planned or unplanned, what kind of invasive measures were needed, and an indication of the physiological state of the patient on arrival in the ICU. Furthermore, it is population-based, relatively large, and has longer follow-up than comparable studies.

This study has several limitations. Although data were registered prospectively, the retrospective study design carries inherent risks of missing or misclassified information. The multicentre setting, although a strength, may also have introduced heterogeneity, as ICU admission criteria may vary between hospitals. Some centres may also use high-dependency or enhanced observation units that are not captured in the registries, potentially biasing the results. Data on adjuvant treatment were largely missing, limiting conclusions regarding its impact on long-term survival. Preoperative status was assessed only by age and ASA grade, the latter being a subjective measure, although it has been validated as a predictor of surgical morbidity and mortality in several studies^40^. No nutritional markers (for example albumin, prealbumin) or detailed co-morbidity data (such as diabetes, chronic obstructive pulmonary disease, cardiovascular disease) were available. Finally, unlike some comparable registries, the SCRCR does not record respiratory complications, despite these being a frequent cause of postoperative deterioration and ICU admission in both this and other cohorts.

## Supplementary Material

zraf178_Supplementary_Data

## Data Availability

Anonymous original data may be provided upon request.
