# Supplementary material for: Unplanned intensive care unit admission after elective colon cancer resection: population-based registry study
Source: BJS Open. 2026 Mar 9;10(2):zraf178. doi: 10.1093/bjsopen/zraf178 (PMC12971005; doi:10.1093/bjsopen/zraf178)
Supplement: zraf178_Supplementary_Data [file zraf178_supplementary_data.docx]

**Unplanned intensive care unit admission after elective colon cancer resection: Population-based registry study**

Sofia Dahlberg^1^ MD PhD, Tomas Vedin^2^ MD PhD, Ingvar Syk^2^ MD PhD, Emma Larsson^3^ MD PhD, Niklas Nielsen^4^ MD Professor, Henrik Bergenfeldt^5^ MD PhD

^1^ Department of Surgery, Helsingborg Hospital and Department of Clinical sciences, Lund, Department of Surgery, Lund University

^2^ Department of Surgery, Skåne University Hospital, Malmö and Institution of Clinical Sciences Malmö, Department of Surgery, Lund University

^3^ Department of Perioperative Medicine and Intensive Care, Karolinska University Hospital and Department of Physiology and Pharmacology, Karolinska Institutet, Stockholm

^4^ Department of Anaesthesiology, Helsingborg Hospital and Department of Clinical sciences, Lund University

^5^ Department of Surgery, Skåne University Hospital, Lund and Department of Clinical sciences Lund, Department of Surgery, Lund University

**Corresponding author**: Sofia Dahlberg, Department of Surgery, Charlotte Yhléns gata 10, 251 87 Helsingborg, Sweden. Sofia.irma.dahlberg@gmail.com

**Supplementary Materials - Index**

| **Supplementary Figures and Tables** |  |
| --- | --- |
| **Table S1.** Factors associated with unplanned postoperative ICU admission, estimated by multivariable logistic regression | *page 2* |
| **Table S2.** Factors associated with 30-day mortality, estimated by multivariable logistic regression  **STROBE Statement -** Checklist of items that should be included in reports of cohort studies. | *page 3*  *page 4 - 5* |
|  |  |
|  |  |

**Supplementary Figures and Tables**

**Table S1.** Factors associated with unplanned postoperative ICU admission, estimated by multivariable logistic regression

| **Unplanned postoperative ICU admission** | **Univariable logistic regression** | | **Multivariable logistic regression** | |
| --- | --- | --- | --- | --- |
|  | **OR** | **95 % CI** | **OR** | **95% CI** |
| Age | 1.035 | 1.29 – 1.041 | 1.029 | 1.021 – 1.037 |
| Male Sex | 1.47 | 1.31 – 1.64 | 1.093 | 0.96 – 1.25 |
| ASA class  1  2  3  4 | Reference  2.24  5.10  10.59 | 1.68 – 2.99  3.83 – 6.79  7.44 – 15.053 | Reference  1.47  2.40  4.74 | 1.068 – 2.018  1.73 – 3.31  3.14 – 7.16 |
| BMI > 30 | 1.49 | 1.32 – 1.69 | 1.24 | 1.063 – 1.44 |
| Perioperative bleeding >500 ml | 2.41 | 2.11 – 2.75 | 1.54 | 1.30 – 1.82 |
| Laparoscopic surgery | 0.49 | 0.43 – 0.56 | 0.59 | 0.50 – 0.69 |
| Perioperative perforation | 2.097 | 1.45 – 3.024 | 1.55 | 0.98 – 2.44 |
| Operating time | 1.0024 | 1.0019 – 1.0029 | 1.0019 | 1.0013 – 1.0025 |
| Cardiovascular complication | 11.20 | 9.51 – 13.18 | 6.60 | 5.39 – 8.081 |
| Infectious complication | 6.67 | 5.80 – 7.67 | 3.51 | 2.95 – 4.18 |
| Neurological complication | 4.59 | 2.79 – 7.55 | 2.049 | 1.10 – 3.81 |
| Surgical complication | 6.0029 | 5.35 – 6.74 | 1.44 | 1.19 – 1.76 |
| Other complication | 4.82 | 4.19 – 5.55 | 2.33 | 1.95 – 2.77 |
| Surgical re-intervention | 12.89 | 11.41 – 14.57 | 8.068 | 6.58 – 9.89 |
| Tumor stage | 1.011 | 0.94 – 1.087 | 0.94 | 0.86 – 1.023 |

**Table S2.** Factors associated with 30-day mortality, estimated by multivariable logistic regression

| **30-day mortality** | **Univariable logistic regression** | | **Multivariable logistic regression** | |
| --- | --- | --- | --- | --- |
|  | **OR** | **95 % CI** | **OR** | **95% CI** |
| Age | 1.086 | 1.070 – 1.10 | 1.064 | 1.045 – 1.083 |
| Male Sex | 1.47 | 1.17 – 1.84 | 1.27 | 0.97 – 1.67 |
| ASA class  1  2  3  4 | Reference  2.77  9.78  26.94 | 1.28 – 5.98  4.59 – 20.83  11.93 – 60.86 | Reference  1.20  2.23  3.23 | 0.53 – 2.72  0.99 – 5.020  1.31 – 7.98 |
| BMI > 30 | 0.96 | 0.73 – 1.27 | 0.82 | 0.59 – 1.14 |
| Perioperative bleeding >500 ml | 1.97 | 1.51 – 2.58 | 1.36 | 0.96 – 1.93 |
| Laparoscopic surgery | 0.57 | 0.43 – 0.74 | 0.93 | 0.68 – 1.27 |
| Perioperative perforation | 1.56 | 0.69 – 3.53 | 1.0080 | 0.39 – 2.59 |
| Operating time | 1.00 | 0.99 – 1.00 | 1.00 | 0.99 – 1.0010 |
| Cardiovascular complication | 25.40 | 20.019 – 32.23 | 8.044 | 5.99 – 10.81 |
| Infectious complication | 7.68 | 6.011 – 9.82 | 1.85 | 1.34 – 2.55 |
| Neurological complication | 9.079 | 4.67 – 17.66 | 3.79 | 1.63 – 8.81 |
| Surgical complication | 3.91 | 3.10 – 4.93 | 1.25 | 0.84 – 1.84 |
| Other complication | 3.71 | 2.80 – 4.90 | 1.18 | 0.83 – 1.69 |
| Surgical re-intervention | 7.16 | 5.65 – 9.065 | 2.01 | 1.32 – 3.066 |
| Tumor stage | 1.026 | 0.88 – 1.19 | 0.98 | 0.83 – 1.16 |
| Unplanned ICU admission | 27.71 | 21.99 – 34.91 | 7.88 | 5.84 – 10.64 |

|  |  |
| --- | --- |

|  |  |
| --- | --- |

**STROBE Statement -** Checklist of items that should be included in reports of cohort studies.

|  | | **Item No** | **Recommendation** | **Page No** |
| --- | --- | --- | --- | --- |
| Title and abstract | | 1 | (*a*) Indicate the study’s design with a commonly used term in the title or the abstract | 1 |
|  |  |  | (*b*) Provide in the abstract an informative and balanced summary of what was done and what was found | 1 |
| **Introduction** | | | | |
| Background/rationale | | 2 | Explain the scientific background and rationale for the investigation being reported | 3 |
| Objectives | | 3 | State specific objectives, including any prespecified hypotheses | 3 |
| Methods | | | | |
| Study design | | 4 | Present key elements of study design early in the paper | 4-5 |
| Setting | | 5 | Describe the setting, locations, and relevant dates, including periods of recruitment, exposure, follow-up, and data collection | 4-5 |
| Participants | | 6 | (*a*) Give the eligibility criteria, and the sources and methods of selection of participants. Describe methods of follow-up | 4 |
|  |  |  | (*b*) For matched studies, give matching criteria and number of exposed and unexposed | n/a |
| Variables | | 7 | Clearly define all outcomes, exposures, predictors, potential confounders, and effect modifiers. Give diagnostic criteria, if applicable | 5-6 |
| Data sources/ measurement | | 8* | For each variable of interest, give sources of data and details of methods of assessment (measurement). Describe comparability of assessment methods if there is more than one group | 5 |
| Bias | | 9 | Describe any efforts to address potential sources of bias | 6 |
| Study size | | 10 | Explain how the study size was arrived at | 4-5 |
| Quantitative variables | | 11 | Explain how quantitative variables were handled in the analyses. If applicable, describe which groupings were chosen and why | 4-5 |
| Statistical methods | | 12 | (*a*) Describe all statistical methods, including those used to control for confounding | 6 |
|  |  |  | (*b*) Describe any methods used to examine subgroups and interactions | 6 |
|  |  |  | (*c*) Explain how missing data were addressed | 6 |
|  |  |  | (*d*) If applicable, explain how loss to follow-up was addressed | 6 |
|  |  |  | **(*e*) Describe any sensitivity analyses** | **n/a** |
| **Results** | | | |  |
| Participants | | 13* | (a) Report numbers of individuals at each stage of study—eg numbers potentially eligible, examined for eligibility, confirmed eligible, included in the study, completing follow-up, and analysed | 7 |
|  |  |  | (b) Give reasons for non-participation at each stage | 7 |
|  |  |  | (c) Consider use of a flow diagram | 7 |
| Descriptive data | | 14* | (a) Give characteristics of study participants (eg demographic, clinical, social) and information on exposures and potential confounders | 7-8 |
|  |  |  | (b) Indicate number of participants with missing data for each variable of interest | 7-8 |
|  |  |  | (c) Summarise follow-up time (eg, average and total amount) | 8-9 |
| Outcome data | | 15* | Report numbers of outcome events or summary measures over time |  |
| Main results | 16 | (*a*) Give unadjusted estimates and, if applicable, confounder-adjusted estimates and their precision (eg, 95% confidence interval). Make clear which confounders were adjusted for and why they were included | | 8-9 |
|  |  | (*b*) Report category boundaries when continuous variables were categorized | | 8-9 |
|  |  | (*c*) If relevant, consider translating estimates of relative risk into absolute risk for a meaningful time period | | n/a |
| Other analyses | 17 | Report other analyses done—eg analyses of subgroups and interactions, and sensitivity analyses | | 8-9 |
| **Discussion** | | | | |
| Key results | 18 | Summarise key results with reference to study objectives | | 10 |
| Limitations | 19 | Discuss limitations of the study, taking into account sources of potential bias or imprecision. Discuss both direction and magnitude of any potential bias | | 13 |
| Interpretation | 20 | Give a cautious overall interpretation of results considering objectives, limitations, multiplicity of analyses, results from similar studies, and other relevant evidence | | 10-12 |
| Generalisability | 21 | Discuss the generalisability (external validity) of the study results | | 10-12 |
| **Other information** | | | | |
| Funding | 22 | Give the source of funding and the role of the funders for the present study and, if applicable, for the original study on which the present article is based | | 1 |

*Give information separately for exposed and unexposed groups.
